# Nitrogen-Doped Porous Carbon Nanosheets Strongly Coupled with Mo_2_C Nanoparticles for Efficient Electrocatalytic Hydrogen Evolution

**DOI:** 10.1186/s11671-019-3147-z

**Published:** 2019-10-22

**Authors:** Ying Lei, Yong Yang, Yudong Liu, Yaxing Zhu, Mengmeng Jia, Yang Zhang, Ke Zhang, Aifang Yu, Juan Liu, Junyi Zhai

**Affiliations:** 1 CAS Center for Excellence in Nanoscience, Beijing Key Laboratory of Micro-nano Energy and Sensor, Beijing Institute of Nanoenergy and Nanosystems, Chinese Academy of Sciences, Beijing, 100083 China; 2 College of Nanoscience and Technology, University of Chinese Academy of Sciences, Beijing, 100049 China; 3 Department of Materials Science & Engineering, College of Engineering, Peking University, Beijing, 100871 China; 4 College of Environmental Sciences and Engineering, Peking University, Beijing, 100871 China

**Keywords:** Nitrogen-doped porous carbon nanosheet, β-Mo_2_C, Electrocatalyst, Hydrogen evolution reaction

## Abstract

Exploring earth-abundant and noble metal-free catalysts for water electrolysis is pivotal in renewable hydrogen production. Herein, a highly active electrocatalyst of nitrogen-doped porous carbon nanosheets coupled with Mo_2_C nanoparticles (Mo_2_C/NPC) was synthesized by a novel method with high BET surface area of 1380 m^2^ g^−1^ using KOH to activate carbon composite materials. The KOH plays a key role in etching out MoS_2_ to produce Mo precursor; simultaneously, it corrodes carbon to form porous structure and produce reducing gas such as H_2_ and CO. The resulting Mo_2_C/NPC hybrid demonstrated superior HER activity in acid solution, with the overpotential of 166 mV at current density of 10 mA cm^−2^, onset overpotential of 93 mV, Tafel slope of 68 mV dec^−1^, and remarkable long-term cycling stability. The present strategy may provide a promising strategy to fabricate other metal carbide/carbon hybrids for energy conversion and storage.

**Supplementary material:**

**Supplementary information** accompanies this paper at 10.1186/s11671-019-3147-z.

## Introduction

Nowadays, environmental pollution and energy crisis have become the key issues for sustainable development [1, 2]. The key to solving the problem is to achieve a clean and renewable energy source. Hydrogen produced through the decomposition of water by catalysts has been considered as a promising alternative to fossil fuels [3, 4]. Platinum-based catalysts are still the most efficient hydrogen evolution reaction (HER) catalysts to date, but the scarcity and high cost limit their large-scale applications. Therefore, the low-cost and earth-abundant transition metal compounds such as transition metal sulfides [5], oxides [6], nitrides [7], phosphides [8, 9], and carbides [10] appear as substitutions for the noble metal catalysts. Among these electrocatalysts, Mo_2_C has attracted great interest as an efficient HER electrocatalysts since its d-band electronic structure is similar to that of Pt [11]. The catalytic properties of molybdenum carbide mainly rely on the exposure of more active sites and improving the conductivity of the catalysts. Researchers tend to the improvement of the composition and structure of Mo_2_C hybrids; however, the current synthesis of Mo_2_C hybrids mainly require high temperature, which will cause the particles to agglomerate, resulting in a decrease in active surfaces and reducing the HER catalytic performance [12]. In order to diminish the agglomeration of molybdenum carbide, conductive carbon supporting material is normally applied to increase the active surfaces and conductivity. Graphite with a two-dimensional structure proved to be an excellent supporting material for catalyst [13]. Otherwise, high surface area of the catalysts would provide more active sites exposed, thus improving the HER performance. Unfortunately, the recent method for improving the specific surface area of the catalyst is still limited, and researchers tend to make efforts to reduce the size of the catalyst, rarely focusing on increasing the porosity of the material [14, 15]. Therefore, the increase in the specific surface area of Mo_2_C/C composite is restricted. The preparation of porous carbon with high specific surface area (4196 m^2^ g^−1^) from potassium hydroxide activated polymer hydrogel [16] provides a new idea to synthesize the supporting conductive graphite substrate with a porous structure which would provide open space and short diffusion channels for reactants during HER [17]. Previous report has demonstrated that the synergistic effects between Mo_2_C and N dopants in carbon materials would lead to high HER electrocatalytic performance [18]. The controlled synthesis of N-doped porous carbon nanosheets as supporting substrate would possess high surface area, excellent conductivity, high durability, N dopants to enhance electron transfer, and porous structure to promote mass/charge transmission. Moreover, reports have proved that the β-Mo_2_C with a hexagonal structure is the most active phase of the four phases of molybdenum carbide since it has a valence band shape similar to Pt [19]. Thus, it is a challenge to synthesize the nitrogen-doped porous carbon nanosheets coupled with β-Mo_2_C nanoparticles for high-efficiency catalytic hydrogen production.

Herein, we report a novel method of self-template to achieve a highly active and stable noble metal-free electrocatalyst with great porosity. Commercial MoS_2_ was used as Mo source and self-template and dopamine was applied as C and N source, respectively. Since dopamine can easily self-polymerize on the surface of Mo source to form poly-dopamine (PDA) microspheres, it is essential to synthesize catalysts with more active surface exposed to air [20]. Reporters tend to use templates such as SiO_2_ [21] and NaCl [22] to avoid aggregation and form structures with high specific surface area. However, dissolving silica requires hydrofluoric acid, which is a high-risk chemical, and removing the salt template involves more steps. We chose commercial MoS_2_ as Mo source and self-templates since MoS_2_ can react with KOH at high temperature. The removal of the template and the activation of KOH which lead to porous carbon and reducing gas synthesized the final Mo_2_C/NPC hybrid with high catalytic activity. Our synthesis method suggests a promising strategy to fabricate noble metal-free high performance HER catalysts.

## Methods

### Preparation of Mo_2_C/NPC Hybrid and the Reference NPC

In a typical synthesis, 500 mg of commercial MoS_2_ was first dispersed in 100 ml deionized water via sonication process. Then, 120 mg of Trizma® base and 200 mg of dopamine hydrochloride were added into the suspension. The mixture was stirred for 24 h at room temperature, and the product was collected by filter after washed with deionized water. After placing it in oven overnight, the resulting MoS_2_@PDA was carbonized in a tube furnace at 600 °C for 2 h to form MoS_2_@NC. The carbonized MoS_2_@NC was soaked in 4 ml of 7 M KOH, with a KOH to MoS_2_@NC mass ratio of 3:1. The dried KOH/MoS_2_@NC mixture was heated under N_2_ at 800 °C for 1 h. After cooling, the sample was filtered and washed with dilute hydrochloric acid solution and deionized water. It was then dried at 60 °C overnight. The final product was Mo_2_C/NPC, and N-doped porous carbon (NPC) was obtained following a similar procedure except that no commercial MoS_2_ was added.

### Characterization

X-ray diffraction (XRD) was performed on a PANalytical X’Pert3 Powder using Cu Kα radiation (*λ* = 1.54056 Å). The morphology was characterized using a field-emission scanning electron microscopy (SEM, Hitachi SU8020). Transmission electron microscopy (TEM) images and corresponding energy-dispersive X-ray (EDX) elemental mapping images were performed with a FEI Tecnai G2 F20 S-TWIN TMP. Raman spectrum was recorded with a confocal Raman spectrometer (HORIBA LabRAM HR Evolution). X-ray photoelectron spectra (XPS) were carried out on a PHI Quantera-II scanning X-ray microprobe spectrometer with Al Kα radiation (1486.6 eV) as an excitation source. TGA/DSC curve was measured by a TGA/DSC1 Mettler-Toledo thermal analyzer. Specific surface area of the sample was measured with a Micromeritices ASAP 2020 HD88.

### Electrochemical Measurements

All electrochemical tests are conducted with a standard three-electrode system on a CHI660E potentiostat (CH Instruments, China), and all potentials in this paper are referred to reversible hydrogen electrode (RHE) according to E(RHE) = E(Ag/AgCl) + 0.059 pH + 0.197 V. Graphite rod was used as the counter electrode and Ag/AgCl (saturated KCl-filled) as the reference electrode, respectively. A glassy carbon electrode with a diameter of 5 mm covered by 15 μL catalyst ink was used as the working electrode. Typically, in preparation of a working electrode, 4 mg of the Mo_2_C/NPC and 20 μL of Nafion solution are dispersed in 1 mL of 3:1 v/v water/ethanol by ultrasonication for 1 h to form a homogeneous ink. Before the electrochemical tests, the fresh working electrode is cycled 50 times to stabilize the current, and linear sweep voltammetry (LSV) is tested in 0.5 M H_2_SO_4_ at a scan rate of 5 mV s^−1^ without IR compensation. Additionally, cyclic voltammograms (CV) are obtained from 0 to 0.2 V (versus RHE, in 0.5 M H_2_SO_4_) with sweep rates of 20, 40, 60, 80, 100, 120, and 140 mV s^−1^, respectively.

## Results and Discussions

**Fig. 1:**
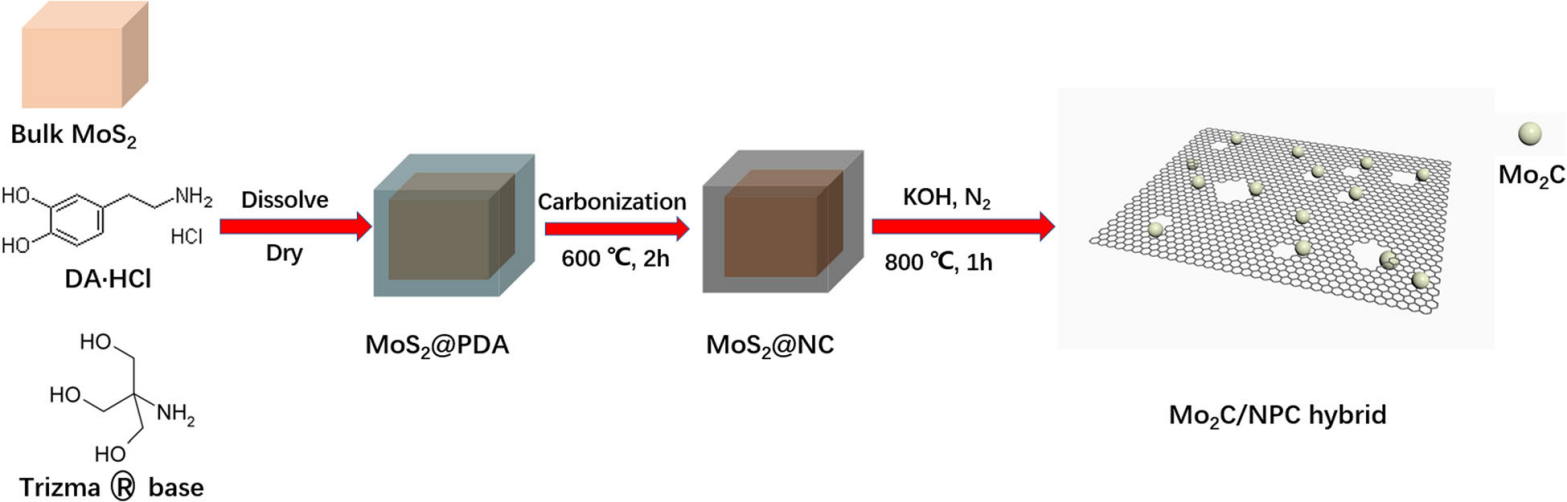
Schematic of the procedure for preparation of Mo_2_C/NPC hybrid

The synthetic procedure of Mo_2_C/NPC hybrid was illustrated in Fig. 1. We chose dopamine as carbon and nitrogen source. Commercial bulk MoS_2_ was selected as Mo source and self-template, by which the size is ~ 2 μm (Additional file 1: Figure S1a). Firstly, the dopamine self-polymerized on the surface of bulk MoS_2_ to form a MoS_2_@PDA core-shell structure (Additional file 1: Figure S1b). Then, the core-shell structure MoS_2_@PDA was carbonized to form N-doped carbon film wrapped on the surface of MoS_2_, which was signed as MoS_2_@NC (Additional file 1: Figure S1c) [23, 24]. Finally, the mixture of the as-prepared MoS_2_@NC and KOH was placed into a tube furnace and reacted to acquire the final product: nitrogen-doped porous carbon nanosheets coupled with Mo_2_C nanoparticles (donated as Mo_2_C/NPC) (Additional file 1: Figure S1d). When MoS_2_ was severed as Mo source, dopamine forms a PDA film on the surface of MoS_2_, the MoS_2_ served as a self-template to avoid dopamine to form microspheres, and a PDA film was generated. This is because the conversion from PDA to N-doped C will continue to maintain its morphology [15]; when MoS_2_ react with KOH, we can get carbon nanosheets about 2 μm in length. The carbon in MoS_2_@NC can also be activated by KOH to get the porous C nanosheets. The formation of Mo_2_C/NPC can be proposed based on a series of reactions. The process of KOH insert and react with carbon can be summarized as KOH activation reaction, the chemical reaction equation is described as 6KOH + 2C ↔ 2K + 3H_2_+ 2 K_2_CO_3_, and the K_2_CO_3_ can be further decomposed into K_2_O, CO_2_, and CO [25]. The process of KOH activation reaction can not only corrode carbon units to produce porous structure of carbon, but also promote the formation of graphitic carbon. Meanwhile, KOH could etch MoS_2_ template to produce Mo_2_C nanoparticles with the diffusion of sulfur vapor and the formation of K_2_S. Thus, the reactions lead to the formation of Mo_2_C/NPC hybrid.

**Fig. 2:**
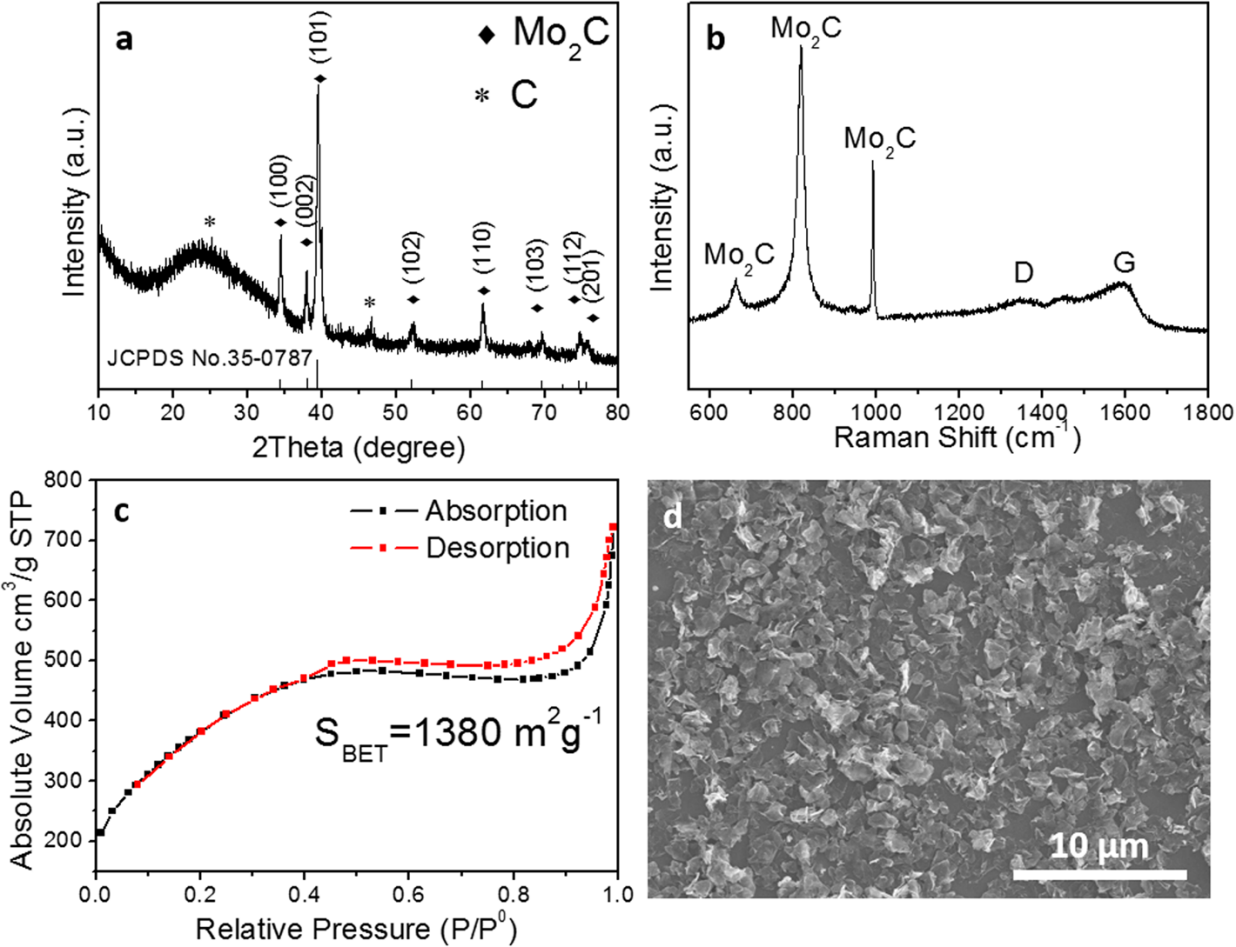
The physical characterization of Mo_2_C/NPC. **a** XRD pattern, **b** Raman spectra, **c** N_2_ adsorption-desorption isotherm, and **d** SEM image

The crystalline phase composition of the product was examined by X-ray diffraction (Fig. 2a). A broad peak near 26°and the peak at 46.3°can be attributed to the (003) and (012) planes of graphitic carbon. The other X-ray diffraction peaks at 34.3, 37.9, 39.39, 52.1, 61.5, 69.5,74.6, and 75.5° are attributed to the diffractions of the (100), (002), (101), (102), (110), (103), (112), and (201) faces of hexagonal β-Mo_2_C (JCPDS 35-0708), respectively. In addition, there are no discernible impurities such as molybdenum metal, oxides, sulfides, or other carbides, indicating the full conversion of commercial MoS_2_ to Mo_2_C. The results of Raman spectroscopy in Fig. 2b further confirmed that the as-prepared catalyst is a mixture of molybdenum carbide and graphite. The intensity ratio of G band to D band, *I*_G_/*I*_D_ > 1, suggests that the carbon is basically graphitic [26]. The amount of Mo_2_C in the final product is found to be ~ 44 wt% based on the thermogravimetric analysis (TGA) in air (Additional file 1: Figure S2). Nitrogen adsorption-desorption isotherms were measured at 77 K to evaluate the Brunauer-Emmett-Teller (BET) specific surface area. As shown in Fig. 2c, the nitrogen adsorption-desorption isotherms of Mo_2_C/NPC showed an H4 type hysteresis loop, which was suitable for materials with micro-meso-pores. Moreover, the BJH desorption average pore size is calculated to be 3.23 nm and the specific BET surface area is 1380 m^2^ g^−1^, which revealed the successful synthesize of the porous structure. Such a micro-meso-porous structure of carbon matrix with ultrahigh surface area is supposed to be an ideal electrode material, which not only can provide open space and short diffusion channels for reactants but can also facilitate the absorption of H^+^ and desorption of H_2_, thus leading to good mass/charge transfer ability.

**Fig. 3:**
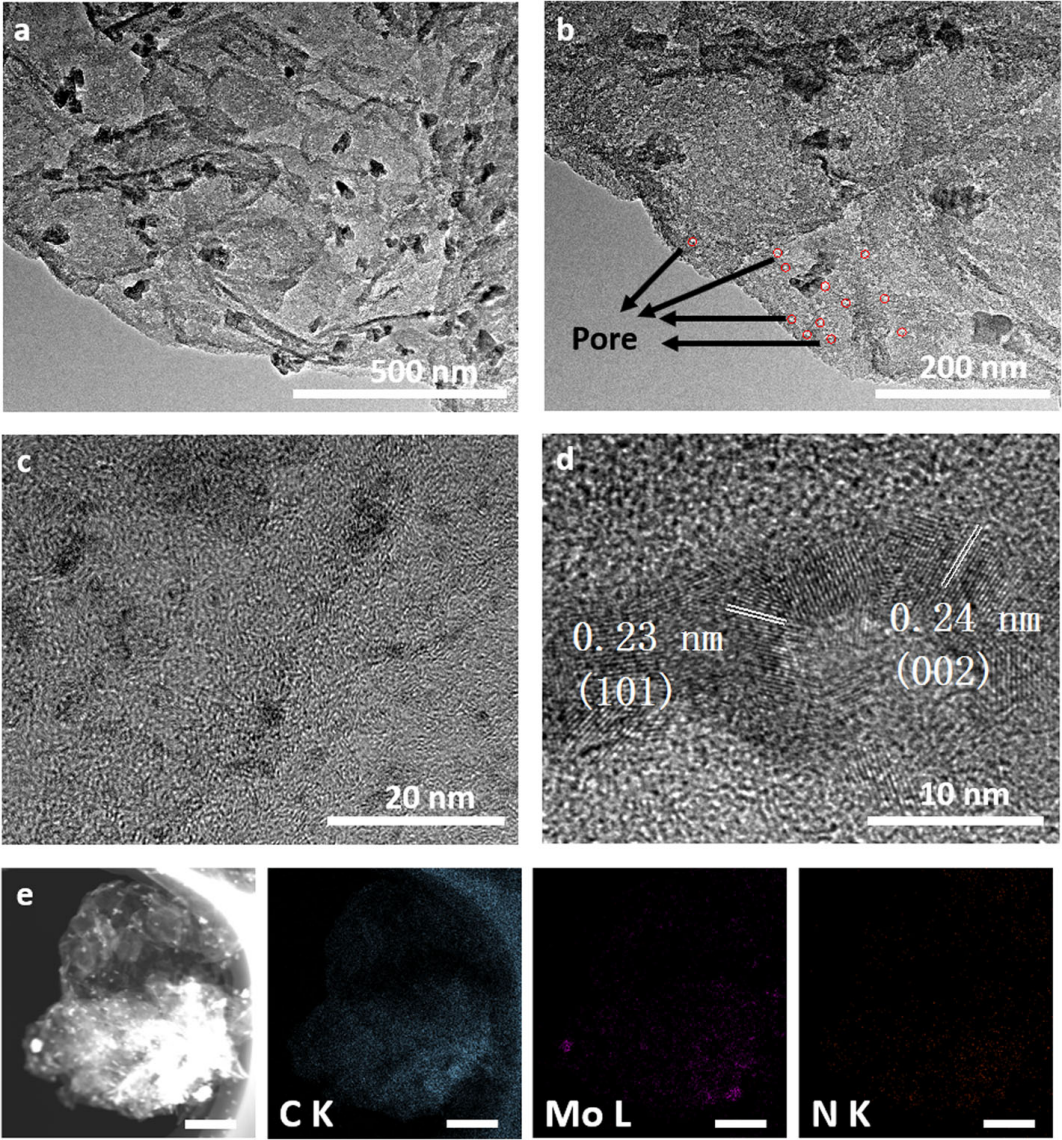
The morphology of Mo_2_C/NPC. **a**–**d** TEM and HRTEM images at different magnifications and **e** corresponding EDS element mapping of Mo_2_C/NPC (scale bar 500 nm)

Then, the morphology and structure of the hierarchical Mo_2_C/NPC hybrid was investigated by SEM and TEM. As shown in Fig. 2d, the low-magnification SEM image presents that numerous well-dispersed nanosheet-like structure with the average size of 2 μm, which is consistent with the size of the template MoS_2_. The TEM images in Fig. 3a and c revealed that the β-Mo_2_C nanoparticles with the size from various several nanometers to 50 nm were anchored on nitrogen-doped carbon nanosheets. The porous nature of carbon nanosheets can be seen from TEM images in Fig. 3b [27]. In addition, high-resolution TEM image in Fig. 3d showed the lattice fringes with d-spacing of 0.23 nm and 0.24 nm which correspond to the (101) and (002) planes of β-Mo_2_C. The porous structure of supporting carbon and the coupling of Mo_2_C nanoparticles with N-doped porous C nanosheets would facilitate the transfer of electrons from molybdenum carbide to carbon, thereby increasing the efficiency of the catalyst. As exemplified in Fig. 3e, the energy dispersive spectroscopy (EDS) analysis demonstrated that the nanosheets were comprised of Mo, C, and N elements, confirming the successful synthesis of Mo_2_C/NPC hybrid.

**Fig. 4:**
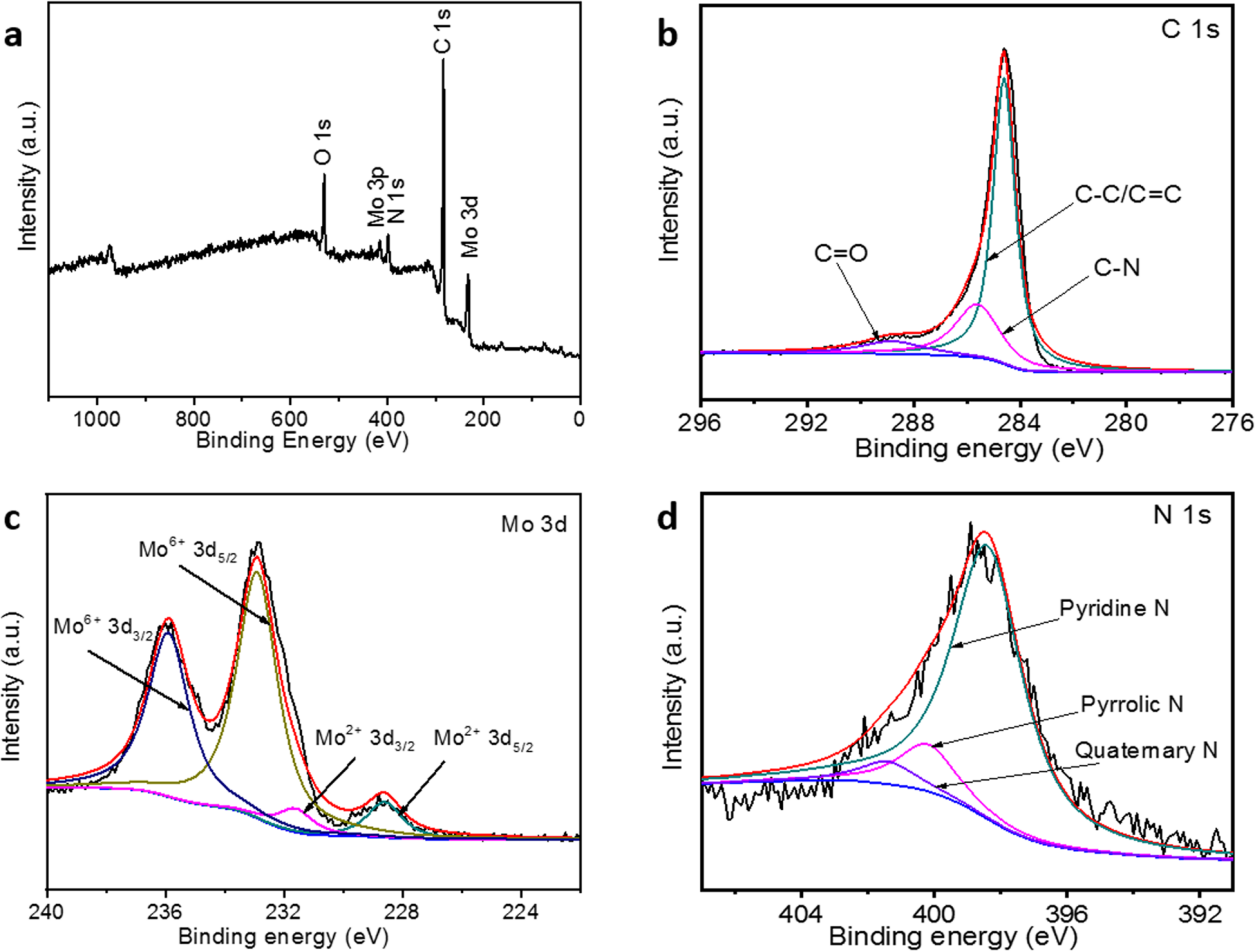
XPS survey spectrum (**a**) and high-resolution XPS scan of C1s (**b**), Mo3d (**c**), and N1 s (**d**) of Mo_2_C/NPC

The surface composition of the as-synthesized Mo_2_C/NPC nanosheets was further elucidated by XPS. From the survey spectrum displayed in Fig. 4a, elements of Mo, C, N, and O can be clearly identified. The C 1s XPS peak can be fitted into three peaks centered at 284.6, 285.6, and 288.8 eV (Fig. 4b), which can be attributed to C-C/C=C, C-N, and C=O species, respectively [28, 29]. The Mo 3d XPS peak can be deconvoluted into two doublets (Fig. 4c). One is centered at binding energy of 228.6/231.6 eV and the other is 232.9/235.9 eV, which can be ascribed to Mo_2_C and surface-oxidized MoO_3_, respectively [14, 26, 29]. The unavoidable abundance of a significant amount of molybdenum oxide is coming from the slow oxidation at the surface of molybdenum carbide when exposed to air [30]. Moreover, it has been reported that the oxide formed on the surface of carbide may retain the activity of the carbide. The N 1s peak (Fig. 4d) at binding energy of 398.4, 400.2, and 401.4 eV can be ascribed to pyridine, pyrrolic, and quaternary N atoms, respectively [24, 29]. Previous report has proved that the N dopants in carbon could induce the electron-transfer process (Mo_2_C→C→N), resulting in a reinforcement of the synergy between Mo_2_C and N dopants in carbon [18].

**Fig. 5:**
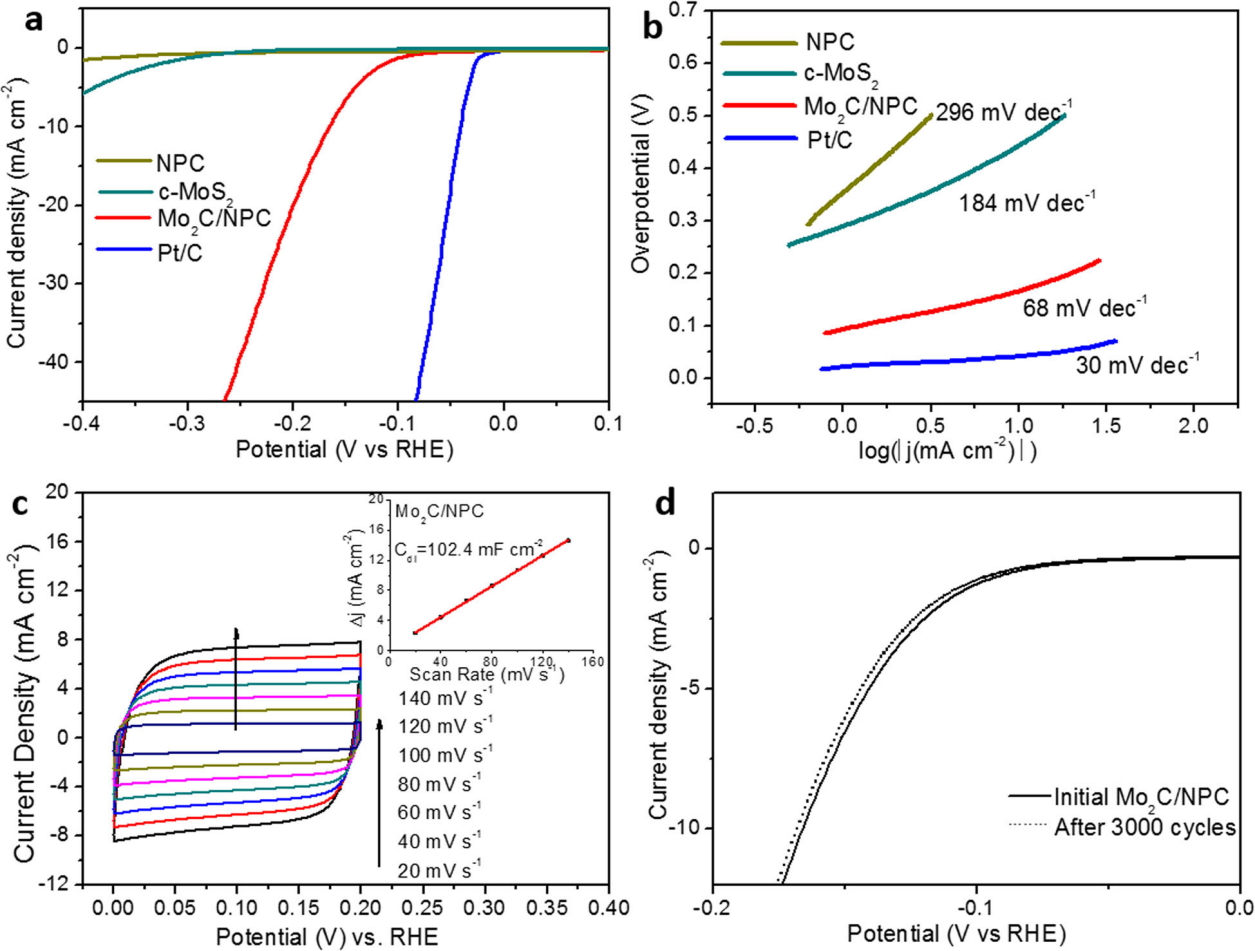
Electrochemical measurements of Mo_2_C/NPC hybrid for HER electrocatalysis in 0.5 M H_2_SO_4_. **a** Polarization curves and **b** Tafel plots of Mo_2_C/NPC in comparison with Pt/C benchmark, c-MoS_2_, and NPC. **c** CV curves of Mo_2_C/NPC under different scan rates from 20 to 140 mV/s. The inset illustrates the plot of capacitive current at 0.1 V against the scan rate. **d** Polarization curves of Mo_2_C/NPC before and after 3000 potential cycles

The electrocatalytic HER activity of the Mo_2_C/NPC was first evaluated in 0.5 M H_2_SO_4_. For comparison, the original commercial MoS_2_ (c-MoS_2_), N-doped porous C (NPC), and 20 wt% Pt/C were also tested by using the same loading amount. Figure 5a compares the corresponding polarization curves. As expected, both the NPC and commercial MoS_2_ showed very limited HER activity, with an onset overpotential of 354 mV and 289 mV, respectively, whereas the Mo_2_C/NPC had an onset overpotential of 93 mV, much lower than that of NPC and c-MoS_2_. The overpotential of the Mo_2_C/NPC at a current density of 10 mA cm^−2^ is 166 mV, much lower than that of NPC and the original c-MoS_2_ and comparable to that of the Mo_2_C/C hybrids in other works [20, 31]. To explore the HER kinetics of the catalysts, Tafel plots were fitted to the Tafel equation (*η* = *a* + *b*log (*j*)), where *b* is the Tafel slope. As shown in Fig. 5b, the Tafel slope of Mo_2_C/NPC was calculated to be 68 mV dec^−1^, much lower than those of c-MoS_2_ (184 mV dec^−1^) and NPC (296 mV dec^−1^), suggesting that the desorption step was efficient on the surfaces of the Mo_2_C/NPC catalysts. The Tafel slope of the Mo_2_C/NPC hybrid falls within the range of 40–120 mV dec^−1^, implying that the HER occurred on the Mo_2_C/NPC surface follows a Volmer-Heyrovsky mechanism [32]. Based on the Tafel analysis, the exchange current density (*j*_0_) of Mo_2_C/NPC was calculated to be 37.4 μA cm^−2^, which outperforms many non-precious HER electrocatalysts reported in the literature (as illustrated in Additional file 1: Table S1) [33–35]. To estimate the electrochemically active surface area (ECSA) of Mo_2_C/NPC under the working conditions, we calculated the double-layer capacitance (*C*_dl_) from cyclic voltammetry (CV) curves at different scan rates in Fig. 5c. As shown in the inset of Fig. 5c, the linear correlation of the current density at 0.1 V against the scan rate indicated that the *C*_dl_ of Mo_2_C/NPC is 102.4 mF cm^−2^. If we assume a standard value of 60 μF/cm^2^, the ECSA of Mo_2_C/NPC is estimated to be ∼ 558 m^2^/g (the calculation is shown in Additional file 1: Figure S3). Such a high ECSA is contributed from both Mo_2_C and the carbon support. Since carbon is much lighter, the N-doped porous C is estimated to account for the most ECSA [26] and it is consistent with the specific BET surface area, thus supports that most of the active Mo_2_C surface is electrochemically accessible.

Besides the HER activity, the stability is another decisive factor to evaluate a catalyst. Long-term cyclic voltammetry was conducted to measure the stability of the Mo_2_C/NPC in 0.5 M H_2_SO_4_. The HER polarization curves in Fig. 5d for the Mo_2_C/NPC show only 2 mV loss after 3000 cycles, indicating the negligible stability of the catalyst. The chronoamperometric response curve of Mo_2_C/NPC at the overpotential of − 0.166 V vs. the RHE was illustrated in the Additional file 1: Figure S4. Based on the above electrochemical study, the remarkable electrocatalytic performance of Mo_2_C/NPC nanosheets can be ascribed to the following factors: (1) the high specific surface area of the catalysts would lead to more active sites for H^+^ absorption, and the good conductivity of the supporting substrate would improve the electron transmission; (2) the coupling of β-Mo_2_C nanoparticles and N-doped porous C nanosheets would enlarge the contact of the catalyst with the electrolyte, facilitating the charge and mass transfer; and (3) the doping N atoms can not only better interact with H^+^ than C atoms but also modify the electronic structures of adjacent Mo and C atoms, making Mo_2_C/NPC a highly efficient catalyst.

## Conclusions

In summary, a novel strategy for preparing hierarchical Mo_2_C/NPC hybrid was developed through a KOH activation method. Commercial MoS_2_ was used as Mo source and self-template while dopamine was used as C and N source. MoS_2_ was etching out by KOH to produce Mo precursor, and the carbonized PDA was corroded by KOH to form porous graphite substrate. The excellent HER activity of Mo_2_C/NPC hybrid with the overpotential of 166 mV at 10 mA cm^−2^, the onset overpotential of 93 mV, Tafel slope of 68 mV dec^−1^, and outstanding long-term cycling stability is attributed to the nitrogen doping content, the porous conductive substrate, the abundance of active sites, and the strong interaction between Mo_2_C and graphitic carbon. This effective method may be applied to the design and preparation of other carbide compounds with high specific surface area for various electrocatalytic applications.

## Supplementary information

**Additional file 1: Figure S1.** SEM images of (a) c-MoS_2_, (b) MoS_2_@PDA, (c) MoS_2_@C and (d) Mo_2_C/NPC. **Figure S2.** TGA curve of the as-prepared Mo_2_C/NPC nanosheets. **Figure S3.** The calculation of ECSA for Mo_2_C/NPC. **Figure S4.** Chronoamperometric response at the potential of -0.166 V vs. the RHE. **Table S1.** Comparison of the exchange current density (j_0_) in acidic media for Mo_2_C/NPC with other non-noble metal electrocatalysts. (DOCX 932 kb)

## Data Availability

All data are fully available without restriction.

## Abbreviations

c-MoS_2_: Commercial MoS_2_
HER: Hydrogen evolution reaction
HRTEM: High-resolution transmission electron microscopy
Mo_2_C/NPC: Nitrogen-doped porous carbon nanosheets coupled with Mo_2_C nanoparticles
MoS_2_@NC: Nitrogen-doped carbon film wrapped on the surface of MoS_2_
NPC: Nitrogen-doped porous carbon
PDA: Poly-dopamine
Pt/C: Platinum/carbon catalyst
RHE: Reversible hydrogen electrode
TGA: Thermogravimetric analysis

## References

[1] Yu F, Zhou H, Huang Y, Sun J, Qin F, Bao J, Goddard WA, Chen S, Ren Z (2018). High-performance bifunctional porous non-noble metal phosphide catalyst for overall water splitting. Nat Commun.

[2] Tan Chaoliang, Luo Zhimin, Chaturvedi Apoorva, Cai Yongqing, Du Yonghua, Gong Yue, Huang Ying, Lai Zhuangchai, Zhang Xiao, Zheng Lirong, Qi Xiaoying, Goh Min Hao, Wang Jie, Han Shikui, Wu Xue-Jun, Gu Lin, Kloc Christian, Zhang Hua (2018). Preparation of High-Percentage 1T-Phase Transition Metal Dichalcogenide Nanodots for Electrochemical Hydrogen Evolution. Advanced Materials.

[3] Kibsgaard J, Chen Z, Reinecke BN, Jaramillo TF (2012). Engineering the surface structure of MoS2 to preferentially expose active edge sites for electrocatalysis. Nat Mater.

[4] Chen Zhijie, Duan Xiaoguang, Wei Wei, Wang Shaobin, Ni Bing-Jie (2019). Recent advances in transition metal-based electrocatalysts for alkaline hydrogen evolution. Journal of Materials Chemistry A.

[5] Deng J, Li H, Wang S, Ding D, Chen M, Liu C, Tian Z, Novoselov KS, Ma C, Deng D, Bao X (2017). Multiscale structural and electronic control of molybdenum disulfide foam for highly efficient hydrogen production. Nat Commun.

[6] Zhang T, Wu M-Y, Yan D-Y, Mao J, Liu H, Hu W-B, Du X-W, Ling T, Qiao S-Z (2018). Engineering oxygen vacancy on NiO nanorod arrays for alkaline hydrogen evolution. Nano Energy.

[7] Wei B, Tang G, Liang H, Qi Z, Zhang D, Hu W, Shen H, Wang Z (2018). Bimetallic vanadium-molybdenum nitrides using magnetron co-sputtering as alkaline hydrogen evolution catalyst. Electrochem Commun.

[8] Yang J, Zhang F, Wang X, He D, Wu G, Yang Q, Hong X, Wu Y, Li Y (2016). Porous molybdenum phosphide nano-octahedrons derived from confined phosphorization in UIO-66 for efficient hydrogen evolution. Angew Chem Int Ed.

[9] Xu H, Wei J, Zhang K, Shiraishi Y, Du Y (2018). Hierarchical NiMo phosphide nanosheets strongly anchored on carbon nanotubes as robust electrocatalysts for overall water splitting. ACS Appl Mater Interfaces.

[10] Fan X, Liu Y, Peng Z, Zhang Z, Zhou H, Zhang X, Yakobson BI, Goddard WA, Guo X, Hauge RH, Tour JM (2017). Atomic H-induced Mo2C hybrid as an active and stable bifunctional electrocatalyst. ACS nano.

[11] Chen WF, Wang CH, Sasaki K, Marinkovic N, Xu W, Muckerman JT, Zhu Y, Adzic RR (2013). Highly active and durable nanostructured molybdenum carbide electrocatalysts for hydrogen production. Energy Environ Sci.

[12] Alhajri N, Anjum D, Takanabe K (2014). Molybdenum carbide-carbon nanocomposites synthesized from a reactive template for electrochemical hydrogen evolution. J Mater Chem A.

[13] Deng D, Novoselov KS, Qiang F, Zheng N, Tian Z, Bao X (2016). Catalysis with two-dimensional materials and their heterostructures. Nat Nanotech.

[14] Chen YY, Zhang Y, Jiang WJ, Zhang X, Dai Z, Wan LJ, Hu JS (2016). Pomegranate-like N,P-Doped Mo2C@C nanospheres as highly active electrocatalysts for alkaline hydrogen evolution. ACS nano.

[15] Chen L, Jiang H, Jiang H, Zhang H, Guo S, Hu Y, Li C (2017). Mo-based ultrasmall nanoparticles on hierarchical carbon nanosheets for superior lithium ion storage and hydrogen generation catalysis. Advanced Energy Materials.

[16] He J, To JWF, Psarras PC, Yan H, Atkinson T, Holmes RT, Nordlund D, Bao Z, Wilcox J (2016) Tunable polyaniline-based porous carbon with ultrahigh surface area for CO2 capture at elevated pressure. Adv Energy Mater 6

[17] Fu Jing, Hassan Fathy M., Zhong Cheng, Lu Jun, Liu Han, Yu Aiping, Chen Zhongwei (2017). Defect Engineering of Chalcogen-Tailored Oxygen Electrocatalysts for Rechargeable Quasi-Solid-State Zinc-Air Batteries. Advanced Materials.

[18] Liu Y, Yu G, Li GD, Sun Y, Asefa T, Chen W, Zou X (2015). Coupling Mo2C with nitrogen-rich nanocarbon leads to efficient hydrogen-evolution electrocatalytic sites. Angew Chem Int Ed.

[19] Wan C, Regmi YN, Leonard BM (2014). Multiple phases of molybdenum carbide as electrocatalysts for the hydrogen evolution reaction. Angew Chem Int Ed.

[20] Wang C, Sun L, Zhang F, Wang X, Sun Q, Cheng Y, Wang L (2017) Formation of Mo-polydopamine hollow spheres and their conversions to MoO2 /C and Mo2 C/C for efficient electrochemical energy storage and catalyst. Small 13 10.1002/smll.201701246 28692790

[21] Chen W, Pei J, He CT, Wan J, Ren H, Zhu Y, Wang Y, Dong J, Tian S, Cheong WC, Lu S, Zheng L, Zheng X, Yan W, Zhuang Z, Chen C, Peng Q, Wang D, Li Y (2017). Rational design of single molybdenum atoms anchored on N-doped carbon for effective hydrogen evolution reaction. Angew Chem Int Ed.

[22] Meng Tao, Zheng Lirong, Qin Jinwen, Zhao Di, Cao Minhua (2017). A three-dimensional hierarchically porous Mo2C architecture: salt-template synthesis of a robust electrocatalyst and anode material towards the hydrogen evolution reaction and lithium storage. Journal of Materials Chemistry A.

[23] Sun X, Jiang J, Yang Y, Shan Y, Gong L, Wang M (2019). Enhancing the performance of Si-based photocathodes for solar hydrogen production in alkaline solution by facilely intercalating a sandwich N-doped carbon nanolayer to the interface of Si and TiO2. ACS Appl Mater Interfaces.

[24] Fan Z, Ding B, Guo H, Shi M, Zhang Y, Dong S, Zhang T, Dou H, Zhang X (2019). Dual dopamine derived polydopamine coated N-doped porous carbon spheres as a sulfur host for high-performance lithium-sulfur batteries. Chemistry.

[25] Zhang J, Zhang C, Zhao Y, Amiinu IS, Zhou H, Liu X, Tang Y, Mu S (2017). Three dimensional few-layer porous carbon nanosheets towards oxygen reduction. Appl Catal B Environ.

[26] Huang Y, Gong Q, Song X, Feng K, Nie K, Zhao F, Wang Y, Zeng M, Zhong J, Li Y (2016). Mo2C nanoparticles dispersed on hierarchical carbon microflowers for efficient electrocatalytic hydrogen evolution. ACS nano.

[27] Jia J, Xiong T, Zhao L, Wang F, Liu H, Hu R, Zhou J, Zhou W, Chen S (2017). Ultrathin N-doped Mo2C nanosheets with exposed active sites as efficient electrocatalyst for hydrogen evolution reactions. ACS nano.

[28] Wang C, Zhang K, Xu H, Du Y, Goh MC (2019). Anchoring gold nanoparticles on poly(3,4-ethylenedioxythiophene) (PEDOT) nanonet as three-dimensional electrocatalysts toward ethanol and 2-propanol oxidation. J Colloid Interface Sci.

[29] Lu C, Tranca D, Zhang J, Rodri Guez Hernandez FN, Su Y, Zhuang X, Zhang F, Seifert G, Feng X (2017). Molybdenum carbide-embedded nitrogen-doped porous carbon nanosheets as electrocatalysts for water splitting in alkaline media. ACS nano.

[30] Ma R, Zhou Y, Chen Y, Li P, Liu Q, Wang J (2015). Ultrafine molybdenum carbide nanoparticles composited with carbon as a highly active hydrogen-evolution electrocatalyst. Angew Chem Int Ed.

[31] Wu Z-Y, Hu B-C, Wu P, Liang H-W, Yu Z-L, Lin Y, Zheng Y-R, Li Z, Yu S-H (2016). Mo2C nanoparticles embedded within bacterial cellulose-derived 3D N-doped carbon nanofiber networks for efficient hydrogen evolution. NPG Asia Mater.

[32] Shi Z, Nie K, Shao Z-J, Gao B, Lin H, Zhang H, Liu B, Wang Y, Zhang Y, Sun X, Cao X-M, Hu P, Gao Q, Tang Y (2017). Phosphorus-Mo2C@carbon nanowires toward efficient electrochemical hydrogen evolution: composition, structural and electronic regulation. Energy Environ Sci.

[33] Jie D, Qiang W, Huang C, Yao W, Xu Q (2018). Cost Effective Mo Rich Mo2C Electrocatalysts for hydrogen evolution reaction. J Mater Chem A.

[34] Cui W, Cheng N, Liu Q, Ge C, Asiri AM, Sun X (2014). Mo2C nanoparticles decorated graphitic carbon sheets: biopolymer-derived solid-state synthesis and application as an efficient electrocatalyst for hydrogen generation. ACS Catal.

[35] Wu HB, Xia BY, Yu L, Yu XY, Lou XW (2015). Porous molybdenum carbide nano-octahedrons synthesized via confined carburization in metal-organic frameworks for efficient hydrogen production. Nat Commun.

