# Supplementary material for: Nitrogen-Doped Porous Carbon Nanosheets Strongly Coupled with Mo2C Nanoparticles for Efficient Electrocatalytic Hydrogen Evolution
Source: Nanoscale Res Lett. 2019 Oct 22;14:329. doi: 10.1186/s11671-019-3147-z (PMC6805847; doi:10.1186/s11671-019-3147-z)
Supplement: Supplementary file 1 — Additional file 1: Figure S1. SEM images of (a) c-MoS2, (b) MoS2@PDA, (c) MoS2@C and (d) Mo2C/NPC. Figure S2. TGA curve of the as-prepared Mo2C/NPC nanosheets. Figure S3. The calculation of ECSA for Mo2C/NPC. Figure S4. Chronoamperometric response at the potential of -0.166 V vs. the RHE. Table S1. Comparison of the exchange current density (j0) in acidic media for Mo2C/NPC with other non-noble metal electrocatalysts. (DOCX 932 kb) [file 11671_2019_3147_MOESM1_ESM.docx]

**Electronic Supporting Information**

**Nitrogen-doped porous carbon nanosheets strongly coupled with Mo_2_C nanoparticles for efficient electrocatalytic hydrogen evolution**

Ying Lei,^1,2^ Yong Yang,^3^ Yudong Liu,^1,2^ Yaxing Zhu,^1,2^ Mengmeng Jia,^1,2^ Yang Zhang,^1,2^ Ke Zhang,^1,2^ Aifang Yu^1,2^, Juan Liu^4*^ and Junyi Zhai^1,2*^

^1^ CAS Center for Excellence in Nanoscience, Beijing Key Laboratory of Micro-nano Energy and Sensor, Beijing Institute of Nanoenergy and Nanosystems, Chinese Academy of Sciences, Beijing 100083, China.

^2^ College of Nanoscience and Technology, University of Chinese Academy of Sciences, Beijing 100049, China

^3^ Department of Materials Science & Engineering, College of Engineering, Peking University, Beijing 100871, China.

^d^ College of Environmental Sciences and Engineering, Peking University, Beijing, 100871, China

Email: jyzhai@binn.cas.cn


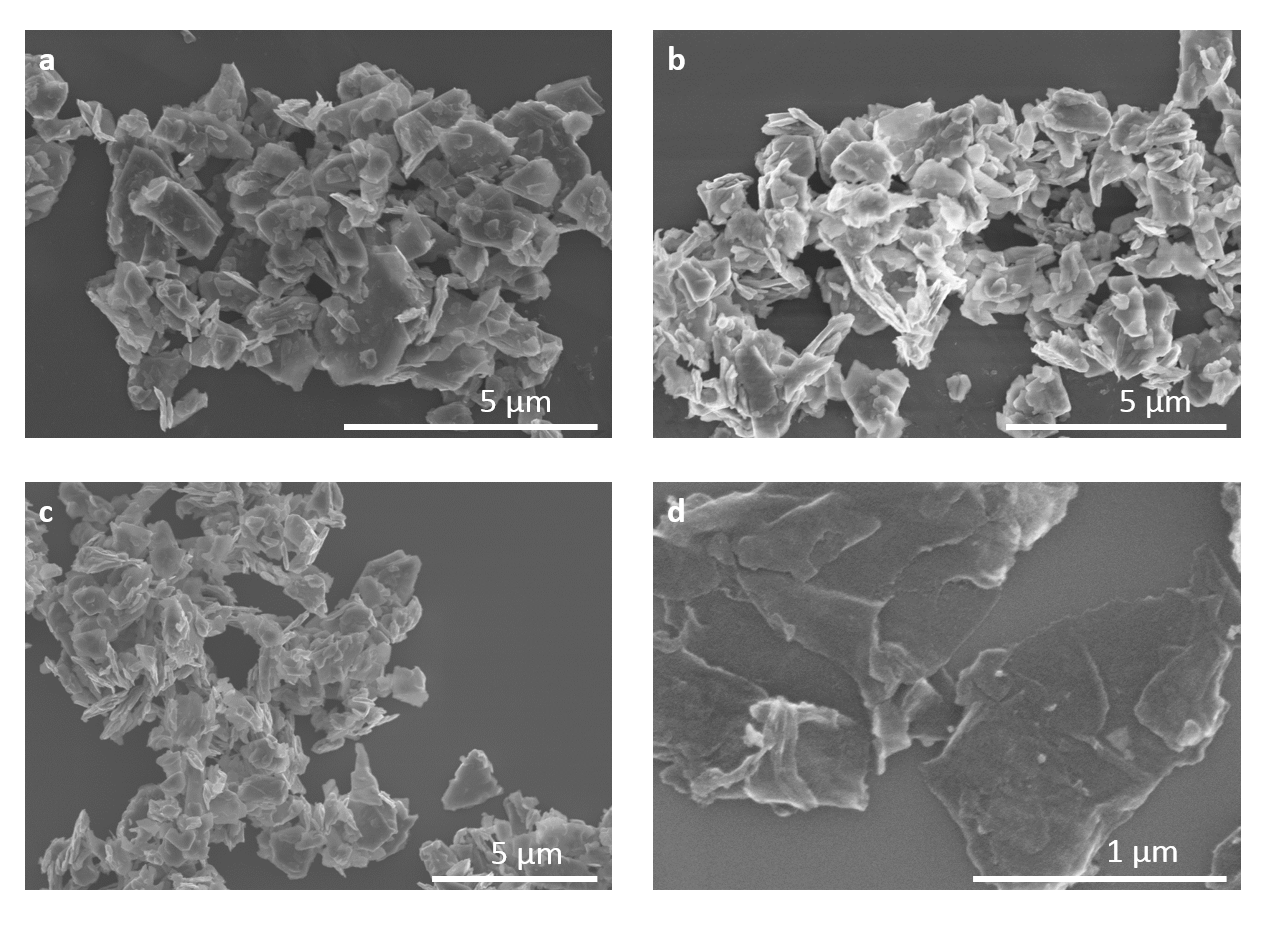


**Fig. S1.** SEM images of (a) c-MoS_2_, (b) MoS_2_@PDA, (c) MoS_2_@NC and (d) Mo_2_C/NPC.

**Fig. S2.** TGA curve of the as-prepared Mo_2_C/NPC nanosheets from 100 to 700 ℃ under air gas flow with a temperature ramp of 10 ℃ min^-1^.

For the TGA curve, the initial weight gain below 350 ℃ is due to the gradual oxidation of Mo_2_C to MoO_3_, followed by a significant weight loss caused by the combustion of carbon.

The remaining weight of the sample after heating to 600 ℃ is about 62 wt.%. Therefore, the Mo_2_C content could be calculated from the following equation:

m (Mo_2_C) = 62 wt.%*M(Mo_2_C)/2M(MoO_3_) = 62 wt.%*204/288 ≈ 44 wt.%

**Fig. S3.** The calculation of ECSA for Mo_2_C/NPC.

Loading amount of the catalyst is 0.305 mg cm^-2^, we assume a standard value of 60 μF/cm^2^, C_dl_ of the catalyst is 102.4 mF cm^-2^.

C=C_dl_/m=335 F/g

ECSA=C/(60 μF cm^-2^)=558 m^2^/g


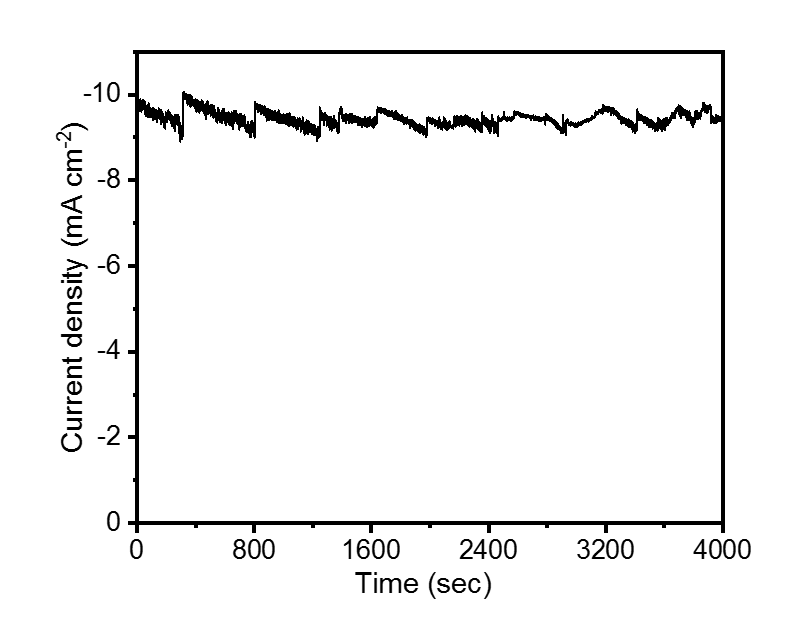


**Fig. S4.** Chronoamperometric response at the potential of -0.166 V vs. the RHE.

**Tab. S1.** Comparison of the exchange current density (j_0_) in acidic media for Mo_2_C/NPC with other non-noble metal electrocatalysts.

| Catalysts | j_0_ (μA cm^-2^) | Ref. |
| --- | --- | --- |
| Mo_2_C/NPC | 37.4 | This work |
| nw-W4MoC | 29 | [[1](#_ENREF_1)] |
| Mo_2_C Nanotubes | 17 | [[2](#_ENREF_2)] |
| Mo_2_C/CNT | 14 | [[3](#_ENREF_3)] |
| Mo_2_C/GCSs | 12.5 | [[4](#_ENREF_4)] |
| β-Mo_2_C-20 | 33 | [[5](#_ENREF_5)] |
| Mo–Mo_2_C-0.077 | 19 | [[6](#_ENREF_6)] |
| MoC_x_ | 23 | [[7](#_ENREF_7)] |

**References**

1. Xiao P, Ge X, Wang H, Liu Z, Fisher A, and Wang X (2015) Novel Molybdenum Carbide-Tungsten Carbide Composite Nanowires and Their Electrochemical Activation for Efficient and Stable Hydrogen Evolution*.* Adv Funct Mater 25:1520-1526

2. Ma F X, Wu H B, Xia B Y, Xu C Y, and Lou X W (2015) Hierarchical β-Mo2 C Nanotubes Organized by Ultrathin Nanosheets as a Highly Efficient Electrocatalyst for Hydrogen Production*.* Angew Chem Int Ed 54:15395

3. Chen W F, Wang C H, Sasaki K, Marinkovic N, Xu W, Muckerman J T, Zhu Y, and Adzic R R (2013) Highly active and durable nanostructured molybdenum carbide electrocatalysts for hydrogen production*.* Energy Environ Sci 6:943

4. Cui W, Cheng N, Liu Q, Ge C, Asiri A M, and Sun X (2014) Mo2C Nanoparticles Decorated Graphitic Carbon Sheets: Biopolymer-Derived Solid-State Synthesis and Application as an Efficient Electrocatalyst for Hydrogen Generation*.* ACS Catal 4:2658-2661

5. Tang C, Sun A, Xu Y, Wu Z, and Wang D (2015) High specific surface area Mo 2 C nanoparticles as an efficient electrocatalyst for hydrogen evolution*.* Journal of Power Sources 296:18-22

6. Jie D, Qiang W, Huang C, Yao W, and Xu Q (2018) Cost Effective Mo Rich Mo2C Electrocatalysts for Hydrogen Evolution Reaction*.* J Mater Chem A 10.1039.C1038TA02550A

7. Wu H B, Xia B Y, Yu L, Yu X Y, and Lou X W (2015) Porous molybdenum carbide nano-octahedrons synthesized via confined carburization in metal-organic frameworks for efficient hydrogen production*.* Nat Commun 6:6512
